# Resistance Genes and their Interactions with Bacterial Blight/Leaf Streak Pathogens (*Xanthomonas oryzae*) in Rice (*Oryza sativa* L.)—an Updated Review

**DOI:** 10.1186/s12284-019-0358-y

**Published:** 2020-01-08

**Authors:** Nan Jiang, Jun Yan, Yi Liang, Yanlong Shi, Zhizhou He, Yuntian Wu, Qin Zeng, Xionglun Liu, Junhua Peng

**Affiliations:** 1 Southern Regional Collaborative Innovation Center for Grain and Oil Crops in China, College of Agronomy, Hunan Agricultural University, Changsha, 410128 Hunan China; 2 Huazhi Rice Bio-tech Company Ltd., Changsha, 410125 Hunan China; 3 Key Laboratory of Coarse Cereal Processing, Ministry of Agriculture Rural Affairs, School of Pharmacy and Bioengineering, Chengdu University, Chengdu, 610106 Sichuan China

**Keywords:** Rice, *Xanthomonas oryzae*, Bacterial blight, Bacterial leaf streak, *R* genes, TAL effector

## Abstract

Rice (*Oryza sativa* L.) is a staple food crop, feeding more than 50% of the world’s population. Diseases caused by bacterial, fungal, and viral pathogens constantly threaten the rice production and lead to enormous yield losses. Bacterial blight (BB) and bacterial leaf streak (BLS), caused respectively by gram-negative bacteria *Xanthomonas oryzae* pv. *oryzae* (*Xoo*) and *Xanthomonas oryzae* pv. *oryzicola* (*Xoc*), are two important diseases affecting rice production worldwide. Due to the economic importance, extensive genetic and genomic studies have been conducted to elucidate the molecular mechanism of rice response to *Xoo* and *Xoc* in the last two decades. A series of resistance (*R*) genes and their cognate avirulence and virulence effector genes have been characterized. Here, we summarize the recent advances in studies on interactions between rice and the two pathogens through these *R* genes or their products and effectors. Breeding strategies to develop varieties with durable and broad-spectrum resistance to *Xanthomonas oryzae* based on the published studies are also discussed.

## Background

Plants are always attacked by diverse and widespread potential pathogens, which cause numerous diseases. These diseases lead to 16% of global crop yield losses (Oerke 2006). Plants have evolved sophisticated innate ability of each cell to fend off the attack (Spoel and Dong 2012). There are two-layered system involved in plant immune response. The first layer is governed by cell surface-localized pattern recognition receptors (PRRs) that detect pathogen-associated molecular patterns (PAMPs), such as bacterial flagellin or fungal chitin, which are highly conserved molecules essential for the pathogen’s life cycle, and trigger a relatively weak immunity (PTI). PTI comprises a wide array of responses, including the production of reactive oxygen species (ROS), increases in intracellular calcium concentration, callose deposition in cell wall, antimicrobial compounds called phytoalexins and the activation of mitogen-activated protein kinases (MAPKs) (Leach et al. 2014). It is a broad-spectrum resistance that wards off most invading organisms. To counter PTI, the pathogens evolved mechanisms to secret and deliver highly variable effectors into host cells to suppress PTI, which is called effector-triggered susceptibility (ETS). The second layer of plant defense acts largely inside the cell and is based on highly polymorphic resistance proteins which directly or indirectly recognize specific virulence effectors secreted within host cells by pathogens, inducing the effector-triggered immunity (ETI). ETI is a rapid and stronger resistance response, usually associated with programmed cell death at sites of infection, termed the hypersensitive response (HR). Other defense responses include the production of ROS, enhancement of cell walls, accumulation of toxic metabolites or proteins, and altered levels of hormone (Leach et al. 2014).

The ancient domesticated crop, rice (*Oryza sativa* L.) is the most important staple food for humans and is one of the most widely cultivated crops all over the world (Ainsworth 2008). Though rice production has been almost doubled over the recent decades due to the introduction of the semi-dwarf gene *sd1*, hybrids, and improvements in cultivation management practices, it needs to significantly increase in order to meet the projected demand from the ever-expanding human population (Khush 2005; Skamnioti and Gurr 2009). However, the increase is challenged by farmland availability, water, soil fertility, climate change, insects and diseases. Rice is vulnerable to a number of diseases caused by bacteria, viruses, or fungi (Dai et al. 2010). Rice bacterial blight (BB) and bacterial leaf streak (BLS) are caused by gram negative bacteria *Xanthomonas oryzae* pv. *oryzae* (*Xoo*) and *Xanthomonas oryzae* pv. *oryzicola* (*Xoc*), respectively. BB is one of the most devastating rice diseases, which can cause severe yield loss of up to 50% depending on the rice variety, growth stage, the geographic location and environmental conditions (Liu et al. 2014). Losses due to the kresek syndrome of BB can reach as much as 75% (Ou 1985). BLS is another devastating rice disease which could spread rapidly under favourable conditions and cause tremendous damage. Yield losses due to BLS range from 8%–32% (Liu et al. 2014). It is becoming more and more important, especially in Asia and Africa. In China, quarantine regulations are now in force for BLS (Li and Wang 2013). In this updated review, we provide an overview of these two diseases and summarize the advances in studies on the *Xoo*/*Xoc*-rice interaction. We also discuss strategies for breeding broad-spectrum and durable disease-resistant rice varieties.

### Overview of the Pathogens and Diseases

BB is one of the oldest recorded rice diseases, which was first found by a farmer in the Fukuoka area of southern Japan in 1884 (Nino-Liu et al. 2006). Since then, it was observed in other regions of Japan and gradually spread to all the rice-growing areas of this country. In China, rice BB was observed as early as 1930s and it spread throughout ten provinces in the south of China by the end of 1950s. However, rice BB was not a severe disease until the 1970s (Zhang 2009). Damage caused by this disease was significantly increased due to the widespread cultivation of semi-dwarf and hybrid rice varieties, as well as massive input of nitrogen fertilizer. It was prevalent in other Asian countries during this period, including India, Philippines, Nepal, Indonesia and Sri Lanka. After that, its incidence was reported in Australia, America and West Africa. To date, rice BB is widely distributed in almost all the rice-growing countries in the world (Naqvi 2019).

BLS was first observed in Philippines in 1918. Since then, the occurrence of BLS in the tropical and subtropical Asia, northern Australia and West Africa was also reported. In China, it was first observed in Guangdong Province, and has recently become one of the major diseases in South China (Tang et al. 2000; Xie et al. 2014).

**Fig. 1:**
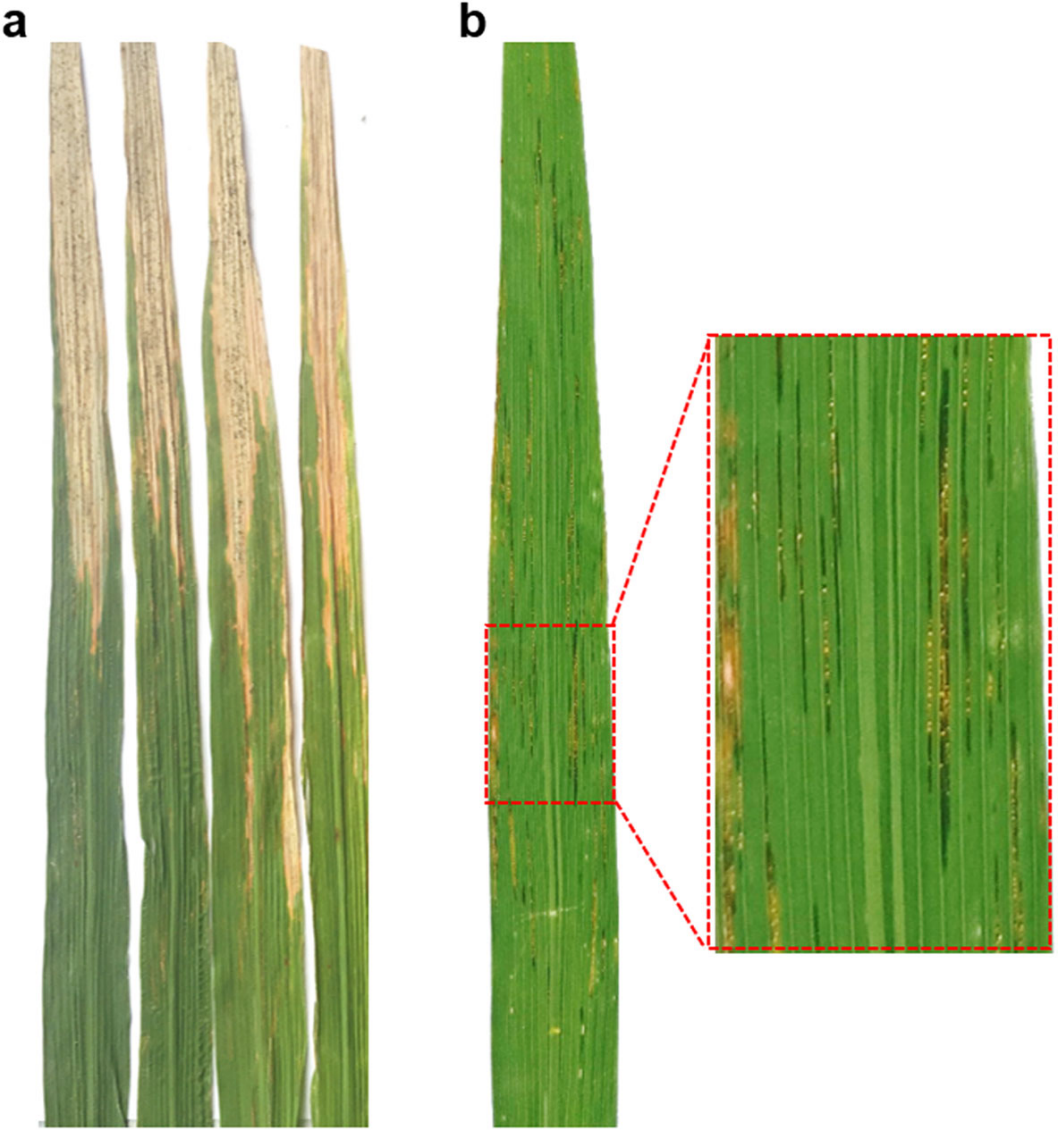
Symptoms of (**a**) bacterial light caused by *Xanthomonas oryzae* pv. *oryzae* and (**b**) bacterial leaf streak caused by *Xanthomonas oryzae* pv. *oryzicola*

Though *Xoc* and *Xoo* are highly related bacterial species, they infect rice in different ways. *Xoo* enters leaf through the hydathodes or wounds, multiplies in the intercellular spaces of the underlying epitheme, and propagate to reach the xylem vessels. The bacteria move through the veins of leaves and spread into the plant. Water-soaked spots at the leaf tips and margins were first observed. Then, the leaves become chlorotic and necrotic along the leaf veins (Lee et al. 2011) (Fig. 1a). *Xoc* penetrates the leaf mainly through stomata or wounds, multiplies in the substomatal cavity and then colonizes the intercellular spaces of the parenchyma. Different from BB, small, water-soaked lesions anywhere along the leaf between the veins were observed during the early stage of BLS infection, resulting in translucent and yellow streaks (Fig. 1b). The infected leaves turn greyish white and die later on (Nino-Liu et al. 2006).

Diverse effector proteins with virulence, avirulence functions or both are secreted by *Xanthomonas oryzae*. Among them, transcription activator like (TAL) effector proteins are a structurally and functionally distinct class of proteins secreted into plant cells by a type III secretion (T3S) system. TAL effectors (also termed as TALEs) import in the nucleus and bind to TALE-specific DNA, which is termed as effector binding elements (EBEs). The recognition transcriptionally activates host target genes, resulting in susceptibility or resistance (Bogdanove et al. 2010; Bogdanove and Voytas 2011).

### Disease Resistance Genes and the Interactions

Deployment of gene-conferred host plant resistance provides an economical, effective, environment friendly approach to control plant diseases and minimize the losses. Extensive genetic studies on rice resistance to BB have been conducted over the last 20 years. To date, more than 40 resistance (*R*) genes conferring host resistance to various strains of *Xoo* have been identified and 11 of them were cloned, namely *Xa1*, *Xa3*/*Xa26*, *Xa4*, *xa5*, *Xa10*, *xa13*, *Xa21*, *Xa23*, *xa25*, *Xa27*, and *xa41* (Table 1) (Ji et al. 2018). These *R* genes can be classified into four groups based on their encoding proteins, including receptor-like kinase (RLK) genes (*Xa21*, *Xa3*/*Xa26* and *Xa4*), sugar will eventually be exported transporter (SWEET) genes (*xa13*, *xa25* and *xa41*), executor genes (*Xa10*, *Xa23* and *Xa27*) and other types of genes (*Xa1* and *xa5*). Some of these isolated *R* genes are widely employed in rice breeding programs to control BB, such as *Xa3*/*Xa26* and *Xa4*, which played an important role in controlling the disease in Asia since 1970s. Nearly all the commercial *indica* hybrid rice varieties in China are known to contain *Xa4*, and *Xa3*/*Xa26* is widely distributed in both *indica* and *japonica* varieties in China (Deng et al. 2018; Hu et al. 2017). The cognate avirulence (*Avr*) genes to all the *R* genes except *Xa4* have been reported (Table 1).

**Table 1:** Summary of the cloned rice *R* genes and the cognate *Xanthomonas oryzae Avr* genes

*R* genes		Cognate *Avr* genes		Reference
Gene	Encoding protein	Gene	Encoding protein	
*Xa3*/*Xa26*	LRR-RLK	*AvrXa3*	Unknown	(Sun et al. 2004; Li et al. 2004; Xiang et al. 2006)
*Xa21*	LRR-RLK	*RaxX*	Unknown	(Song et al. 1995; Pruitt et al. 2015)
*Xa4*	Wall-associated kinase/RLK	Not determined	Unknown	(Hu et al. 2017)
*xa13 (OsSWEET11)*	SWEET-type protein	*PthXo1*	TAL effector	(Chu et al. 2006; Yang et al. 2006; Yuan et a. 2012)
*xa25 (OsSWEET13)*	SWEET-type protein	*PthXo2*	TAL effector	(Liu et al. 2011; Zhou et al. 2015)
*xa41 (OsSWEET14)*	SWEET-type protein	*AvrXa7*/*PthXo3*/*TalC/Tal5*	TAL effector	(Antony et al. 2010; Yu et al. 2011; Streubel et al. 2013; Hutin et al. 2015)
*Xa10*	Executor R protein	*AvrXa10*	TAL effector	(Tian et al. 2014)
*Xa23*	Executor R protein	*AvrXa23*	TAL effector	(Wang et al. 2014; Wang et al. 2015)
*Xa27*	Executor R protein	*AvrXa27*	TAL effector	(Gu et al. 2005)
*Xa1*	NLR	*PthXo1*/*Tal4*/*Tal9d*	TAL effector	(Yoshimura et al. 1998; Ji et al. 2016a)
*xa5*	TFIIA transcription factor	*Avrxa5*/*PthXo7*	TAL effector	(Jiang et al. 2006; Zou et al. 2010; Sugio et al. 2007)
*Rxo1*	NLR	*AvrRxo1*	TAL effector	(Zhao et al. 2004a; Zhao et al. 2004b)

*NLR* nucleotide-binding domain and leucine-rich repeat, *LRR-RLK* leucine-rich repeat receptor-like kinase, *TFIIA* transcription factor IIA, *SWEET* sugar will eventually be exported transporter, *TAL* transcription activator like

In contrast to BB, no native major *R* gene controlling resistance to BLS has been identified in rice and only a few of quantitative resistance loci have been mapped. Interestingly, one of them, *qBlsr5a*, with relatively large effect, was mainly controlled by *xa5* (Xie et al. 2014). A non-host *R* gene, *Rxo1*, was isolated from maize, and the transgenic rice with *Rxo1* has been proved to confer high level resistance to BLS (Zhao et al. 2005).

In addition, some defense-related or susceptible genes in rice were reported to be involved in the interaction with *Xoc* (Shen et al. 2010; Tao et al. 2009). Here, we focus on the recent advances in identification of the *R* genes or their products and the cognate pathogen effectors. The underlying molecular mechanisms of the interaction between rice and *Xoo* or *Xoc* are discussed. Additionally, two genes, *Xa7* and *Xo1*, which have not been cloned yet, are also discussed due to their potential value in rice breeding programmes and special features. To date, most of the cloned plant *R* genes encode nucleotide-binding and leucine-rich repeat domain (NLR) proteins (Li et al. 2015).

However, only one encodes NLR protein among the 11 cloned *Xa* genes (Yoshimura et al. 1998). These *Xa* genes are classified into four groups based on the encoded protein types including RLK (receptor-like kinase), SWEET (sugar will eventually be exported transporter), executor R proteins and other proteins.

#### Receptor-Like Kinase (RLK) Genes

In plants, PRRs, which can recognize diverse pathogen-associated molecular patterns are a key component of the innate immune system. All the known plant PRRs are either transmembrane receptor-like kinases (RLKs) or transmembrane receptor-like proteins (RLPs) (Antolín-Llovera et al. 2012). There are over 1100 candidate RLKs/RLPs in rice genome (Shiu et al. 2004). RLKs typically contain an extracellular domain, a single-pass transmembrane domain, and an intracellular kinase domains, whereas RLPs lack the kinase domain (Monaghan and Zipfel 2012). Leucine-rich repeat receptor-like kinases (LRR-RLKs) represent the largest subfamily of plant RLKs (Afzal et al. 2008).

**Fig. 2:**
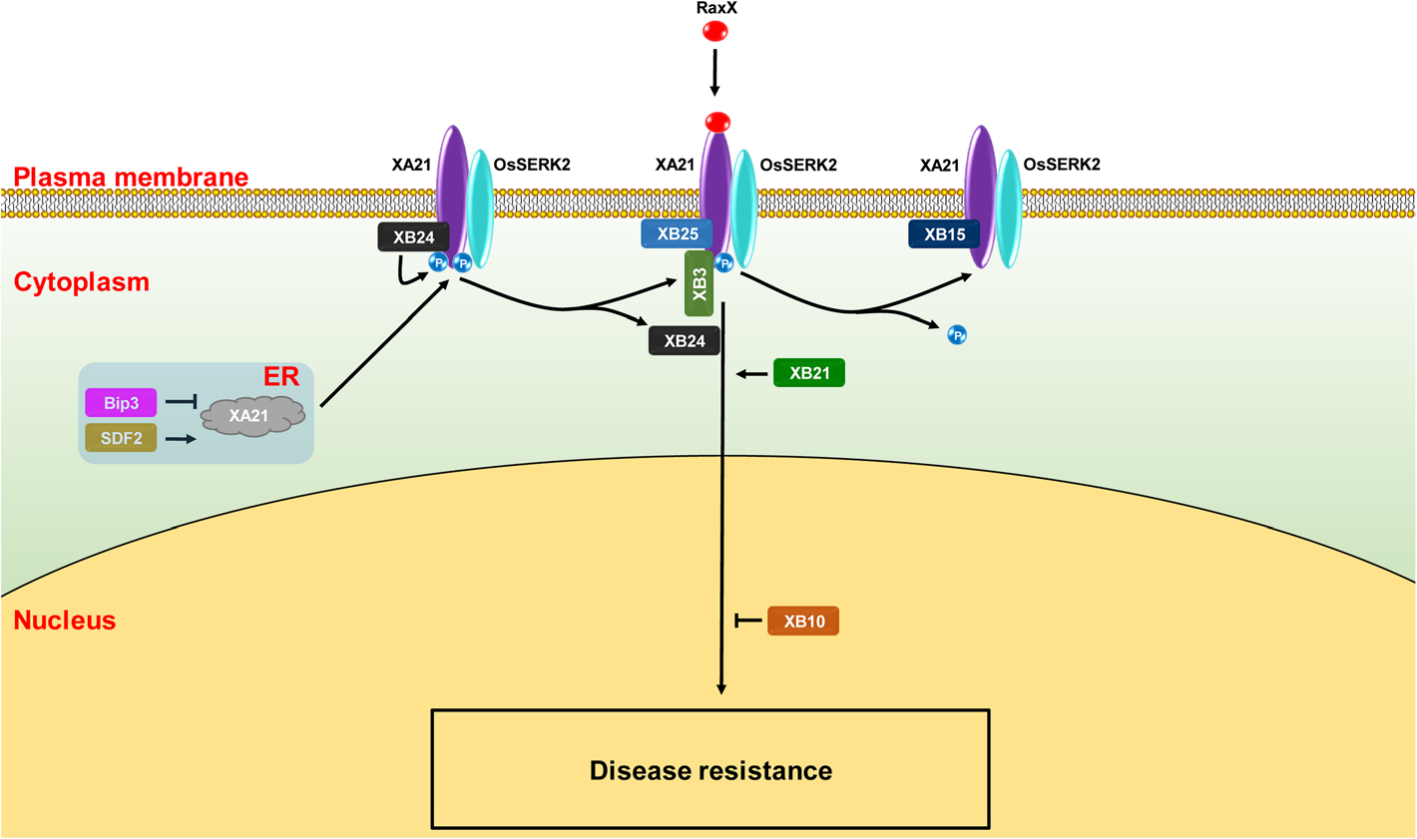
*Xa21*-mediated immune signaling pathways triggered by *Xanthomonas oryzae*. Sulphated RaxX is recognized by XA21 and activate XA21-mediated resistance. Several XA21 binding proteins, including OsSERK2, XB3, XB10, XB15, XB21, XB24, XB25, Bip3 and SDF2 are involved in regulating XA21-mediated resistance. XA21 is processed in endoplasmic reticulum, which is negatively and positively regulated by the ER chaperones BiP3 and SDF2, respectively. OsSERK2 positively regulates the immunity by forming a constitutive complex with XA21 and transphosphorylating XA21. XB24 binds to XA21 and promotes autophosphorylation of XA21 to keep it in an inactive state. During *Xoo* infection, XB24 dissociates from XA21. XB3 and XB25 are required for XA21 accumulation. XB15 dephosphorylates the autophosphorylated XA21 and attenuates the XA21-mediated resistance. XB21 functions as an auxilin to positively regulate XA21-mediated immunity. The transcription factor XB10/OsWRKY62 acts as a negative regulator XA21-mediated immunity

The LRR-RLK gene *Xa21*, originated from the wild rice species *Oryza longistaminata*, was the first cloned *R* gene in rice (Song et al. 1995). *Xa21* have been proved to confer broad-spectrum resistance to *Xoo*. However, *Xa21*-mediated resistance progressively increases from the susceptible juvenile two-leaf stage through later stages, with full resistance only at the adult stage (Century et al. 1999; Wang et al. 1996). Overexpression of *Xa21* gene can enable plants with resistance at both seedling and adult stages (Park et al. 2010a). The regulation of *Xa21*-mediated immunity has been extensively and comprehensively studied. Several XA21 binding proteins (XBs) with diverse functions have been characterized (Table 2, Fig. 2). The phosphorylation state of XA21 is important for its function. In the absence of infection, the ATPase XB24 physically associates with the XA21 juxtamembrane domain and promote phosphorylation of specific serine and threonine residues to maintain the inactive state of the XA21 protein. On recognition of pathogen invasion, the XA21 kinase disassociates from XB24 and triggers downstream defense responses (Chen et al. 2010b). After activation, XB15, a PP2C phosphatase, acts on XA21 and dephosphorylates the autophosphorylated XA21 (Park et al. 2008). The *Xoo* tyrosine-sulfated and type I-secreted protein RaxX is the ligand to induce the XA21-mediated immunity (Pruitt et al. 2015). The sulfated RaxX directly binds XA21 with high affinity (Luu et al. 2019). More details are shown in Table 2 and Fig. 2.

**Table 2:** Summary of XA21-binding proteins

Interacting protein	Gene Locus	Gene product	Subcelluar localization	Role	Function	Reference
XB3	LOC_Os05g02130	RING finger-containing E3 ubiquitin ligase	Not determined	+	Maintains the stability of XA21	(Wang et al. 2006)
XB10	LOC_Os09g25070	Transcription factor	Partially localize to the nucleus	–	Suppresses the activation of defense-related genes	(Peng et al. 2008)
XB15	LOC_Os03g60650	Protein phosphatase 2C	Plasma membrane	–	Dephosphorylates XA21 and attenuates XA21-mediated immune responses	(Park et al. 2008)
XB21	LOC_Os12g36180	Auxilin-like protein	Not determined	+	May function as clathrin uncoating factor to mediate XA21 endocytosis	(Park et al. 2017)
XB24	LOC_Os01g56470	ATPase	Not determined	–	Promotes XA21 autophosphorylation and keep it in a biologically inactive state	(Chen et al. 2010b)
XB25	LOC_Os09g33810	Plant-specific ankyrin-repeat (PANK) protein	Plasma membrane	+	Maintains the stability of XA21	(Jiang et al. 2013; Zhang et al. 2010)
BiP3	LOC_Os02g02410	Heat shock protein (HSP) 70	Endoplasmic reticulum	–	Serves as a XA21 chaperone and regulates XA21 processing	(Park et al. 2010b)
SDF2	LOC_Os08g17680	Stromal-derived factor 2	Endoplasmic reticulum	+	Serves as a XA21 chaperone and regulates XA21 processing	(Park et al. 2013)
	LOC_Os08g34190		Not determined			
OsSERK2	LOC_Os04g38480	Rice somatic embryogenesis receptor kinase 2	Plasma membrane	+	Forms a constitutive complex with XA21 and phosphorylate one another	(Chen et al. 2014)

+, positive impact on XA21-mediated resistance; −, negative impact on XA21-mediated resistance

Another LRR-RLK gene *Xa26*, was originally identified from *indica* variety Minghui 63, an elite restorer line of hybrid rice in China (Sun et al. 2004). Further study demonstrated *Xa3*, identified in a *japonica* variety Wase Aaikoku 3, is the same gene as *Xa26* (Xiang et al. 2006). OsSERK2 and OsTPI1.1 interact with XA3/XA26 and are involved in XA3/XA26-mediated resistance (Chen et al. 2014; Liu et al. 2018). OsTPI1.1 encoding a triosephosphate isomerase (TPI) catalyzes the reversible interconversion of dihydroxyacetone phosphate to glyceraldehyde-3-phosphate. Reduced expression of OsTPI1.1 largely compromises XA3/XA26-mediated resistance. OsTPI1.1 participates in the defense response through TPI which is significantly enhanced by binding with XA3/XA26 (Liu et al. 2018). As well as XA21, XA3/XA26-mediated resistance is positively regulated by OsSERK2 (Chen et al. 2014). *AvrXa3*, the cognate avirulence gene to *XA3/Xa26*, has been isolated, but how it initiates XA3/XA26-meditaed resistance remains unclear (Li et al. 2004).

*Xa4*, encoding a cell wall-associated kinase, confers a race-specific resistance to *Xoo* at all stages of rice growth (Leach et al. 2001; Sun et al. 2003; Hu et al. 2017). Wall-associated kinases (WAKs) are also a subfamily of RLKs that physically link the cell wall with the plasma membrane to transmit extracellular signals to the cytoplasm (Anderson et al. 2001). *Xa4* was first introgressed into commercial rice varieties in the early 1970s. It is one of the most widely employed resistance genes in breeding programs. Nearly all the *indica* hybrid rice cultivars in China carry *Xa4* (Leach et al. 2001). XA4 prevents the invasion of *Xoo* through reinforcing the cell wall (Hu et al. 2017). The accumulation of the two phytoalexins, sakuranetin and momilactone A, which are likely to suppress *Xoo* in plant, is proved to be associated with *Xa4*-mediated resistance. In addition to conferring durable resistance to *Xoo*, *Xa4* increases the mechanical strength of the culm and reduces the plant height slightly, and thus may enhance the lodging resistance (Hu et al. 2017). The multiple favorable agronomic traits related with *Xa4* may explain why it is widely used.

#### Sugar Will Eventually be Exported Transporter (SWEET) Genes

**Fig. 3:**
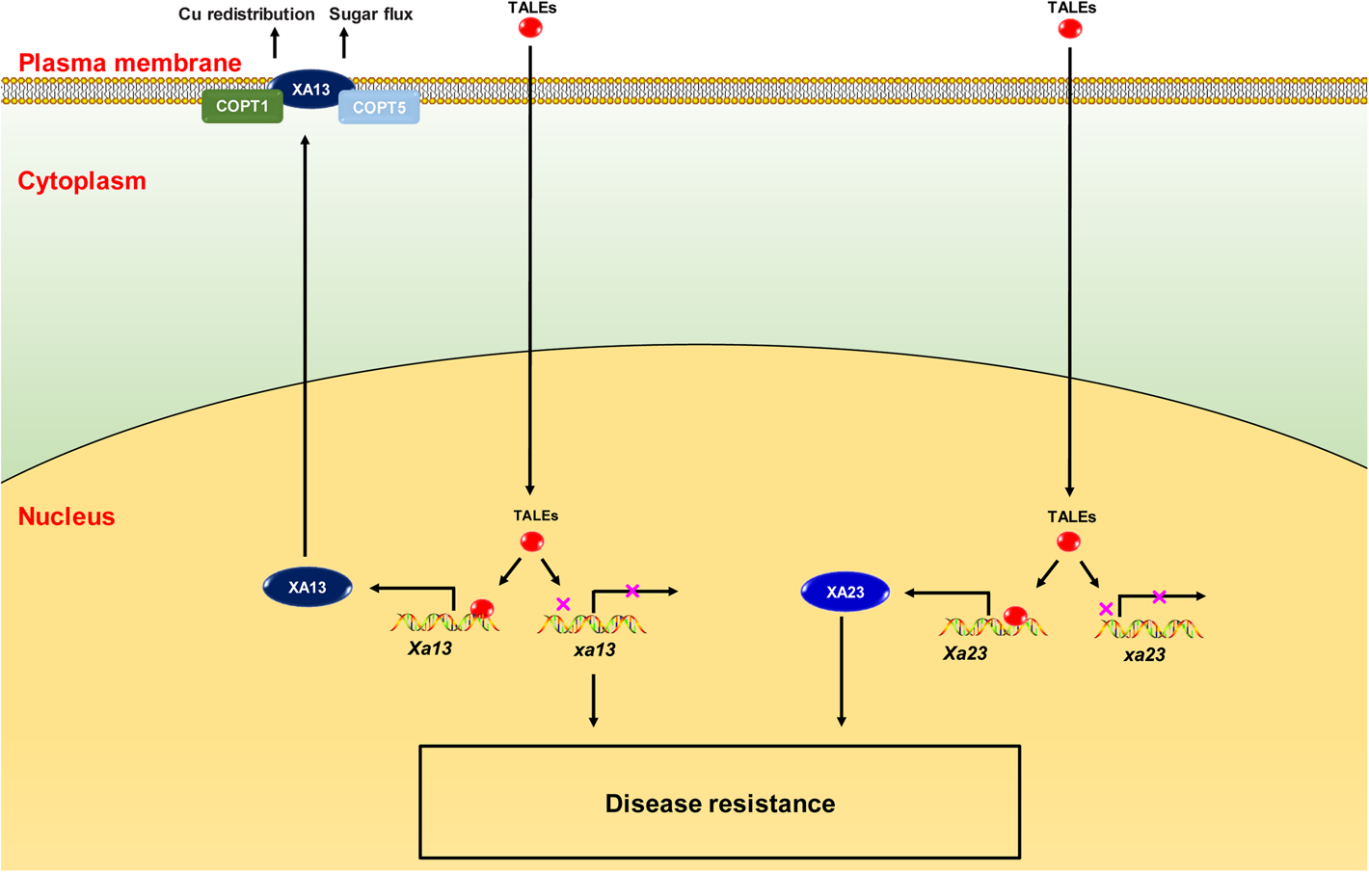
The SWEET gene *xa13* and the executor gene *Xa23* mediated immune signaling pathways triggered by *Xanthomonas oryzae*. The TALEs are secreted into the cytoplasm of plant cells through the type III secretion system, enter the nucleus, bind to the specific promoter elements and induce the expression of *Xa13*. XA13 is hijacked by TALEs to export sucrose to the apoplast, which provides nutrition to the pathogen. XA13 is also employed together with COPT1 and COPT5 by TALEs to remove toxic Cu from xylem vessels. The resistant allele *xa13* with mutations in the EBEs disrupt the binding of TALEs, leading to disease resistance. Like SWEET genes, the executor *R* gene *Xa23* is transcriptionally activated by TALEs, triggering host defense responses

Three recessive *R* genes, *xa13*, *xa25* and *xa41*, encodes clade III SWEET proteins. SWEET, a unique family of sugar efflux transporters, play a vital role in various biological processes, including pollen nutrition, senescence, seed filling and plant-pathogen interactions (Chen et al. 2012; Guan et al. 2008; Quirino et al. 1999; Streubel et al. 2013). SWEETs are grouped into a four-clade phylogenetic tree in plants (Eom et al. 2015). There are 17 and 22 SWEET genes in *Arabidopsis* and rice genomes, respectively (Chen et al. 2010a). Over the last 10 years, several studies have suggested sugar exporting into the apoplast via clade III SWEETs is hijacked by TAL effectors of pathogen, which is essential for pathogen growth and virulence (Eom et al. 2015). The *xa13* (also known as *Os8N3* and *OsSWEET11*) confers specific resistance to *Xoo* race 6, which was originally identified in cultivar BJ1 (Chu et al. 2006). It was isolated through different strategies by two groups (Chu et al. 2006; Yang et al. 2006). The TAL effector PthXo1 from *Xoo* directly targets to the EBEs, in the promoter of dominant *Xa13* but not *xa13* alleles to induce its expression, which is critical for susceptibility (Fig. 3) (Römer et al. 2010; Yuan et al. 2009). Further studies showed that the XA13 protein cooperates with two copper transporters, COPT1 and COPT5, to participate in copper redistribution. Copper is widely used as an important element for pesticides in agriculture. XA13, COPT1 and COPT5 are employed by TAL effectors of *Xoo* and remove toxic Cu from xylem vessels, where pathogen multiplies and spreads to cause disease (Yuan et al. 2010). Interestingly, knock-out of *OsSWEET11* showed increased resistance to *Rhizoctonia solani*, which causes sheath blight disease. It suggests that *OsSWEET11* may also be employed by the fungal pathogen *Rhizoctonia solani* (Gao et al. 2018). In addition, *Xa13* was found to be required for pollen development. The *Xa13*-silenced plants had low fertility, and most pollen grains were defective in comparison with normal pollen grains (Chu et al. 2006).

As well as *Xa3/Xa26*, *xa25* (also known as *OsSWEET13*) was identified from Minghui 63 (Chen et al. 2002). It confers race-specific resistance to *Xoo* strain PXO339 at both seedling and adult stages. Similar to *xa13*, the expression of dominant *Xa25* but not recessive *xa25* was rapidly induced by PXO339 (Liu et al. 2011). Another type of recessive *xa25* alleles was identified in *japonica* rice varieties Nipponbare and Kitaake (Zhou et al. 2015). Further studies showed that *OsSWEET13* as the disease-susceptibility gene is directly targeted by PthXo2. In a very recent study, two types of PthXo2-like TALEs were found to bind with different EBE sequences in the *OsSWEET13* promoter and activate its expression (Xu et al. 2019).

*Xa41* (also known as *Os11N3* and *OsSWEET14*) was found to be targeted as a susceptibility gene by different TAL effectors from numerous *Xoo* strains, including AvrXa7, PthXo3, TalC and Tal5 (Antony et al. 2010; Hutin et al. 2015; Streubel et al. 2013; Yu et al. 2011). A germplasm screening for polymorphisms in the *OsSWEET14* promoter uncovers a natural candidate plant disease resistance gene from African wild and cultivated rice species *O. barthii* and *O. glaberrima* (Hutin et al. 2015). An allele of *OsSWEET14* was identified to carry an 18-bp deletion at 8 bp downstream of the predicted TATA box, and could prevent *OsSWEET14* induction by AvrXa7 and Tal5. The *xa41* confers broad-spectrum resistance to 50% of the tested strains representing genetically distant groups isolated from different countries in Asia and Africa (Hutin et al. 2015). In another study, in silico mining of *OsSWEET13* and *OsSWEET14* promoter polymorphisms in a diversity germplasm panel containing 3000 rice genome sequences and the Pakistani aromatic germplasm collection was conducted (Zaka et al. 2018). Novel variations in the EBEs of *OsSWEET13* and *OsSWEET14* promoter regions were identified (Zaka et al. 2018).

#### Executor Genes

*Xa27*, *Xa10* and *Xa23* are three executor genes with multiple potential transmembrane domains functioning as a promoter trap, which are transcriptionally activated by TAL effectors and trigger defense responses (Gu et al. 2005; Tian et al. 2014; Wang et al. 2015). *Xa27* originated from wild rice *O. minuta* Acc. 101,141 and confers broad-spectrum resistance to *Xoo* strains from different countries (Gu et al. 2005). *Xa27*-mediated resistance is also affected by developmental stage like *Xa21* and *Xa3*/*26*. Challenged by *Xoo* containing *AvrXa27*, *Xa27* was specifically induced and secreted to the apoplast, leading to inhibition of bacterial growth. However, the allele from the susceptible variety IR24 was not induced. Increased expression of *Xa27* showed thickened vascular bundle elements, even in the absence of *Xoo* infection. Further study showed that localization of XA27 to the apoplast depending on the N-terminal signal-anchor-like sequence is important for its resistance to *Xoo* (Wu et al. 2008). Rice lines with both *AvrXa27* and *Xa27* showed enhanced resistance when inoculated with compatible strains of *Xoo* and *Xoc* (Tian and Yin 2009).

*Xa10*, which confers resistance to some Philippine races of *Xoo*, was first identified from rice cultivar Cas 209 (Gu et al. 2008; Lee et al. 2003). *AvrXa10* specifically induces *Xa10* expression through direct binding *Xa10* promoter. Rice plants with constitutive but weak expression of *Xa10* showed lesion mimic phenotype. Further study has revealed that XA10 forms hexamers and locate in the ER membrane of plant and HeLa cells, which mediates the disruption of the ER, cellular Ca^2+^ homeostasis and triggers programmed cell death (Tian et al. 2014).

Another executor gene *Xa23* isolated from a wild rice species of *O. rufipogon*, confers an extremely broad spectrum of resistance to *Xoo* strains isolated from different regions at all growth stages of rice. Similar to *Xa27*, *Xa23* shares identical ORF with the susceptible *xa23* allele, and a 7-bp polymorphism in the promoter regions leads to induction of *Xa23*, but not *xa23*, by *AvrXa23*. Transient expression analysis indicated that XA23 triggers HR in *N. benthamiana* and tomato (Wang et al. 2015). *AvrXa23* was found to be highly conserved in all the tested *Xoo* isolates (Wang et al. 2014). It is possible that AvrXa23 contributes to the virulence of *Xoo* for infection or growth in host plants. The prevalence of *AvrXa23* in natural *Xoo* strains explains why *Xa23* shows the broad-spectrum resistance.

#### Other Genes

In rice genome, 480 nucleotide-binding domain and leucine-rich repeat (NLR) genes have been revealed, but only a single one, *Xa1*, conferring resistance to *Xoo*, was isolated (Yoshimura et al. 1998). *Xa1* was isolated from *japonica* cultivar Kogyoku and its expression was induced by bacterial infection and wounding (Yoshimura et al. 1996). *Xa1* confers resistance against *Xoo* by recognizing several TAL effectors including PthXo1, Tal4 and Tal9d, but truncated interfering TAL effectors (also termed as iTALEs). The iTALEs may function as decoys interfering with the recognition of intact TALEs by XA1 and block its function (Ji et al. 2016a).

The recessive gene *xa5* confers broad resistance spectrum to *Xoo* and is most commonly found in the Aus-Boro varieties from Bangladesh. The *xa5* is a natural allele of *Xa5* for the transcription factor IIA gamma subunit 5 (TFIIAγ5), contains a mutation in the 39th residue, in which the valine (V) residue is replaced with glutamine (E) (V39E) (Jiang et al. 2006). TFIIA is a basal transcription factor of eukaryotes and it is essential for polymerase II-dependent transcription (Høiby et al. 2007). TFIIAγ5 is hijacked by TAL effectors by direct physical interaction with a transcription factor binding (TFB) region of TALEs and attenuate the TALE-associated transcription of host *S* or *R* genes (Yuan et al. 2016). The induction of susceptibility genes, such as *OsSWEET11* and *OsSWEET14* by TALEs are almost abolished in *xa5* background or TFIIAγ5-RNAi transgenic plants, which leads to the improvement of BB resistance. TFIIAγ5 is also necessary for TALE-associated transcription of *R* genes, including *Xa27* and *Xa23*, to defend against disease (Yuan et al. 2016). *Xa27* and *Xa23*-mediated BB resistance are attenuated in the *xa5* background (Gu et al. 2009; Yuan et al. 2016). In the absence *TFIIAγ5*, the other *OsTFIIAγ* gene in rice, *OsTFIIAγ1* plays a compensatory role. Its expression is activated by TALE PthXo7, which increases expression of the host genes (Ma et al. 2018). Interestingly, *TFIIAγ5* is also employed by *Xoc* TALEs to cause disease (Yuan et al. 2016). Mutation and suppression of *TFIIAγ5* can also improve BLS resistance. In another study, *xa5* was found through genetic mapping as a quantitative trait locus with a relatively large effect for resistance to *Xoc* (Xie et al. 2014).

The non-host resistance gene, *Rxo1* encoding a NLR protein, confers high level resistance to *Xoc* in rice. It also controls resistance to the pathogen *Burkholderia andropogonis*, which causes bacterial stripe of sorghum and maize. Transgenic lines with *Rxo1* also showed HR when inoculated with *avrRxo1* containing *Xoc* strain (Zhao et al. 2004a; Zhao et al. 2004b; Zhou et al. 2010). It exhibits the characteristics consistent with those mediated by host resistance genes, activating multiple defensive pathways related to HR. A microarray analysis showed that *Rxo1* functions in the early stage of rice-*Xoc* interaction and involved in signaling pathways leading to HR and some basal defensive pathways such as SA and ET pathways (Zhou et al. 2010).

In addition to the cloned genes above, the dominant *R* gene *Xa7*, which has not been isolated yet, is known for its durable resistance and potential value in rice breeding programmes (Vera Cruz et al. 2000). *Xa7* was originally identified in rice cultivar DV85 and fine mapped to an interval of approximately 118.5 kb on chromosome 6 (Chen et al. 2008). The durable resistance of *Xa7* is due to a fitness penalty in *Xoo* associated with adaptation to *Xa7* (Vera Cruz et al. 2000; Bai et al. 2000). Mutations occurred specifically at the *avrXa7* gene in the adapted strains, which displayed reduced aggressiveness on susceptible rice cultivars (Vera Cruz et al. 2000; Bai et al. 2000). Additionally, *Xa7* are more effective at high temperatures, whereas other *R* genes are less effective (Webb et al. 2010). Another yet uncharacterized gene *Xo1*, was identified in the American heirloom rice variety Carolina Gold Select, and confers resistance to the tested African strains of *Xoc*, but not Asian strains (Triplett et al. 2016). Like *Xa1*, *Xo1-mediated* recognition of full-length TALEs can also be blocked by truncated TALEs (Read et al. 2016). Interestingly, *Xa1* and *Xo1* are located in the same region (Triplett et al. 2016). Further studies are needed to determine whether *Xo1* is controlled by *Xa1* or another gene.

### Breeding Strategies to Develop Broad-Spectrum and Durable Resistance to Xoo and Xoc

Use of host plant resistance is generally the most favorable tactic to control diseases due to economic and environmental reasons. Marker-assisted selection (MAS) and genetic transformation are the two major approaches for *R* gene deployment in plant breeding programs. However, controversy on food safety and constraints on regulatory in some countries have serious plagued the application of genetically modified varieties. MAS, free of political issues and social problems, is more widely used by breeders. Pyramiding *R* genes resistant to different races of the pathogen through marker-assisted breeding strategies, is a very effective way to achieve durable and broad-spectrum resistance, while employment of a single *R* gene and adaption of the pathogen often lead to resistance breakdown in a short period.

Based on the previous reports, *xa5*, *Xa7*, *xa13*, *Xa21* and *Xa23* are more frequently used by rice breeders due to the comparatively broader spectra of resistance. Xu et al. (2012) transferred *Xa7* and *Xa21* into Yihui 1577, an elite hybrid rice restorer line. The pyramiding lines and their derived hybrids displayed resistance to all the seven *Xoo* strains, while the lines containing single *Xa7* or *Xa21* were resistant to six of them. Two Basmati rice varieties PB1121 and PB6 were improved for resistance to BB (*xa13* and *Xa21*) through MAS (Ellur et al. 2016). In another study, three genes, *xa5*, *xa13* and *Xa21* were transferred into Lalat, a popular *indica* variety in Eastern India but susceptible to bacterial blight (Dokku et al. 2013). The improved lines showed significant enhanced resistance.

Because *Xa23* displays broadest resistance, it is often used alone, or along with *R* genes against rice blast disease or/and brown planthopper (Zhou et al. 2011; Huang et al. 2012; Ni et al. 2015; Jiang et al. 2015; Ji et al. 2016b; Xiao et al. 2016). In addition, some gene combinations are ineffective, such as, *xa5* + *Xa23*, *xa5* + *Xa27* (Gu et al. 2009; Yuan et al. 2016). Therefore, deep understanding the underlying molecular mechanisms of *R* gene-mediated resistance is important for its effective application. It is noteworthy that no native major *R* gene effective against *Xoc* has been discovered so far in rice. The recessive *xa5* confers quantitative resistance to BLS, and should be used in combination with other resistance QTL or genes. Fine mapping of previously identified resistance loci with large effect, including the dominant locus *Xo1* and the recessive locus *bls1*, will facilitate employment of them in rice breeding programmes (Triplett et al. 2016; He et al. 2012).

In our breeding practice, we introgressed *Xa7* + *Xa21* into an elite restorer line R900 of hybrid rice through marker-assisted backcrossing (MABC) scheme in less than 3 years, which is much more efficient than the conventional breeding method. The improved lines recovered more than 99% genome background of the recurrent parent R900, and showed a broad-spectrum resistance to *Xoo* without any significant difference in main agronomic traits in both the growth chambers and paddy fields (unpublished data). In addition, the *R* genes can be used separately in time and space. Development of near-isogenic lines and rotation of the *R* genes could reduce the selection pressure on pathogens and maximize the life span of *R* genes. Multi-lines containing different *R* genes also has the potential to provide broad-spectrum and durable disease resistance.

In recent years, the emerging genome-editing technologies, including zinc-finger nucleases (ZFNs), TAL effector nucleases (TALENs) and clustered regularly interspaced short palindromic repeats (CRISPR)/Cas9 (CRISPR-associated protein-9 nuclease), have revolutionized biology by enabling targeted modifications of genomes (Christian et al. 2010; Jinek et al. 2012; Kim et al. 1996). These technologies have been successfully applied in model species *Arabidopsis thaliana*, *Nicotiana benthamiana* and multiple crops including rice, wheat, maize, barley, soyben, tomato, potato, citrus, and sorghum (Shah et al. 2018). The powerful tools have great potential in improving the plant disease resistance. Elimination of EBEs in promoters of susceptibility genes or adding EBEs to promoters of executor *R* genes through genome editing, could enhance the resistance to BLB. For example, the EBEs of AvrXa7 and PthXo3 in the *OsSWEET14* promoter were precisely edited by TALENs, which prevents the induction by TALEs. The mutated lines showed strong resistance to both AvrXa7- and PthXo3-dependent *Xoo* strains (Li et al. 2012). Similarly, the promoter of *Xa13* (*OsSWEET11*) was targeted by CRISPR/Cas9-based disruption, leading to enhanced resistance without affecting rice fertility (Li et al. 2019). In a very recent study, EBEs in the promoters of *OsSWEET11*, *OsSWEET13* and *OsSWEET14* were edited simultaneously by CRISPR/Cas9 technology and rice lines conferring broad-spectrum resistance to *Xoo* were created (Xu et al. 2019). In another study, six EBEs corresponding three TALEs from *Xoo* and three from *Xoc*, were added to the *Xa27* promoter, resulting in broad-spectrum resistance to both *Xoo* and *Xoc* (Hummel et al. 2012). It suggests that engineering of EBEs upstream of rice executor *R* genes through genome-editing technologies is a potential strategy to generate germplasms with broad-spectrum resistance to *Xoo*, *Xoc* and other bacterial pathogens.

## Conclusions

Rice-*Xanthomonas oryzae* patho-system is a powerful model for research toward solutions in disease control. Although tremendous progress has been made in the past decades, there are still many queries and challenges. For example, whether there is any major *R* gene in rice against BLS? The *xa5* confers resistance to both *Xoo* and *Xoc*, does any other identified *Xa* genes have the same effect? The ligand from *Xoo* mediating *Xa4* resistance is still not determined. The partners and/or components associated with *R* proteins remain largely unknown in rice. It will be interesting to understand how *R* genes activate downstream signaling components and trigger plant defense response system. TAL effectors injected into plant cells have to be translocated into nucleus to bind to the target *S* or *R* genes for virulence or plant immunity. However, the underlying mechanism needs to be further elucidated. Altogether, a comprehensive understanding of the molecular interactions between rice and *Xanthomonas oryzae* is the pivotal for more efficient and durable disease control.

## Data Availability

Not applicable.
